# Spontaneous pneumomediastinum associated with Covid-19 pneumonia in a pregnant woman

**DOI:** 10.1590/0037-8682-0185-2021

**Published:** 2021-06-02

**Authors:** Asli Tanrivermis Sayit, Muzaffer Elmali, Mesut Onal

**Affiliations:** 1 Ondokuz Mayis University, Faculty of Medicine, Radiology, Samsun, Turkey.; 2 Ondokuz Mayis University, Faculty of Medicine, Obstetrics and Gynecology, Samsun, Turkey.

**FIGURE 1:**
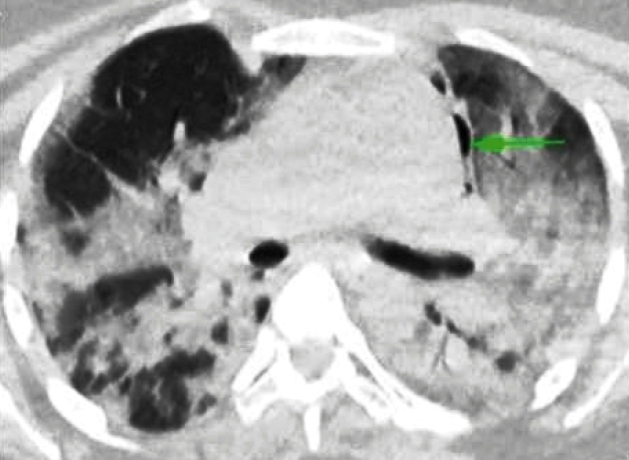
Axial non-enhanced chest computed tomography image revealing diffuse bilateral patchy ground-glass opacities and consolidations in the peripheral and posterior lung regions, with the presence of air content on the left side (arrow), consistent with pneumomediastinum.

**FIGURE 2:**
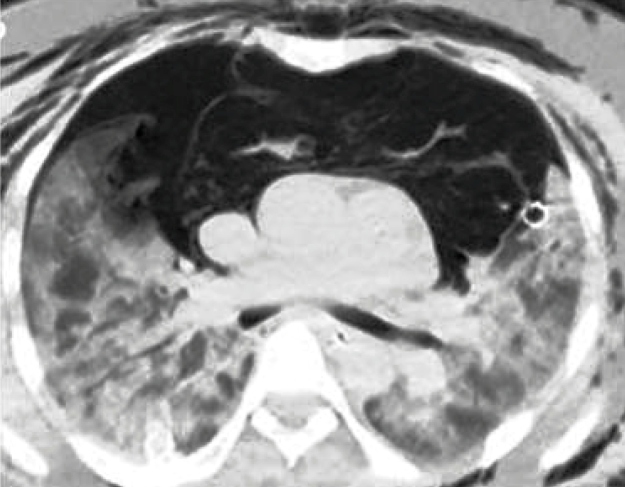
Axial non-enhanced chest computed tomography image revealing diffuse bilateral ground-glass opacities and consolidations in the lungs, pneumomediastinum, bilateral pneumothorax, and excessive subcutaneous emphysema in the chest wall.

A 32-year-old woman presenting with complaints of shortness of breath and cough for 4 days was admitted to the hospital at 28 weeks in her fifth pregnancy. Chest computed tomography (CT) showed diffuse bilateral patchy ground-glass opacities and consolidations in the peripheral and basal lung regions, which were typical findings of coronavirus disease 2019 (Covid-19) pneumonia (Figure 1). Chest CT confirmed left-sided pneumomediastinum. Emergency cesarean section was performed due to acute fetal distress and deterioration of the patient’s general condition. A healthy male infant weighing 1270 g was born. The patient was not extubated after the operation because of low oxygen saturation. On the second postoperative day, crepitus was detected around her neck and chest area. Chest CT confirmed newly developed bilateral pneumothorax and extensive subcutaneous emphysema extending superiorly in the thorax and into the neck and revealed progressive bilateral parenchymal infiltration and pneumomediastinum (Figure 2). A bilateral closed intercostal chest tube was inserted immediately. The patient was extubated after 12 days due to improvement in her general condition and was discharged with oxygen support treatment after 2 months of hospitalization.

Only a few cases of spontaneous pneumothorax and pneumomediastinum in the later stages of Covid-19 pneumonia have been reported^1^ ^-^ ^3^. To the best of our knowledge, this is the first report of spontaneous pneumomediastinum and pneumothorax associated with Covid-19 pneumonia during the second trimester of pregnancy.
